# Anesthetic Management of a Patient With Acute Necrotizing Encephalopathy Type 1

**DOI:** 10.7759/cureus.81291

**Published:** 2025-03-27

**Authors:** Shamim A Beigh, Andrea Yap

**Affiliations:** 1 Anesthesiology, Al Jalila Children's Speciality Hospital, Dubai, ARE

**Keywords:** acute necrotizing encephalopathy, anesthesia, dexmedetomidine, mitochondrial disorders, ran-binding protein 2 mutation

## Abstract

Acute necrotizing encephalopathy (ANE) is a progressive neurodegenerative condition that often arises after exposure to a variety of viruses and systemic infections, including influenza and SARS-CoV-2. It typically presents with developmental delay, seizures, dysarthria, and ataxia. Acute necrotizing encephalopathy type 1 (ANE1) is recurrent, and familial cases have been associated with mutations in the RAN-binding protein 2 (RanBP2) gene. The disease shares symptomatic and pathological resemblance with mitochondrial metabolic disorders. In this case report, we present the anesthetic management of a seven-year-old boy with ANE1 who underwent total hip reconstruction. Literature on the anesthetic management of such patients is sparse, and we discuss the patient's perioperative anesthetic management, including medications, monitoring, and care, along with a literature review.

## Introduction

Acute necrotizing encephalopathy (ANE) was first described by Mizuguchi in 1995 and is an extremely rare and potentially lethal neurological disease. It is characterized by progressive neurodegeneration with the development of symmetrical necrotic lesions in the thalamus, pons, and brainstem that can arise following exposure to a variety of systemic infections, including influenza A and B, parainfluenza 2, and SARS-CoV-2 [1-3]. It usually presents in infancy with seizures, dysarthria, and ataxia with developmental delay. Most of the reported ANE cases are sporadic; however, recurrent and familial cases have been associated with RAN-binding protein 2 (RanBP2) gene mutations [4]. RanBP2, a multifunctional protein, plays a key role in nucleocytoplasmic transport [5-7]. The formation of abnormal RanBP2 can lead to disruptions in nuclear signal and blood-brain barrier function. This subset of ANE patients with RanBP2 gene mutations is categorized as acute necrotizing encephalopathy type 1 (ANE1) [4].

RanBP2 mutations in ANE1 not only have neurological sequelae but also mitochondrial enzyme dysfunction. This leads to impaired glucose metabolism, adenosine triphosphate (ATP) production, and loss of coupling of oxidative phosphorylation [8,9]. This is further compounded by the development of hepatic and renal dysfunction and makes the patient sensitive to the development of hypoglycemia and lactic and metabolic acidosis. RanBP2 mutation can also affect immune function by impairing the ability of mitochondria to regulate the secretion of proinflammatory cytokines, such as IL-1ß and IL-18, thereby altering the cellular response to infection. Unrestrained immune activation results in marked systemic inflammation with deleterious consequences to the host, a condition termed "cytokine storm" [10-15].

Herein, we present the case of a patient with ANE1 disease who required general anesthesia for total hip reconstruction. Due to the rare nature of the disease, there is limited information in the literature regarding the anesthetic and perioperative management of such patients.

## Case presentation

**Figure 1:**
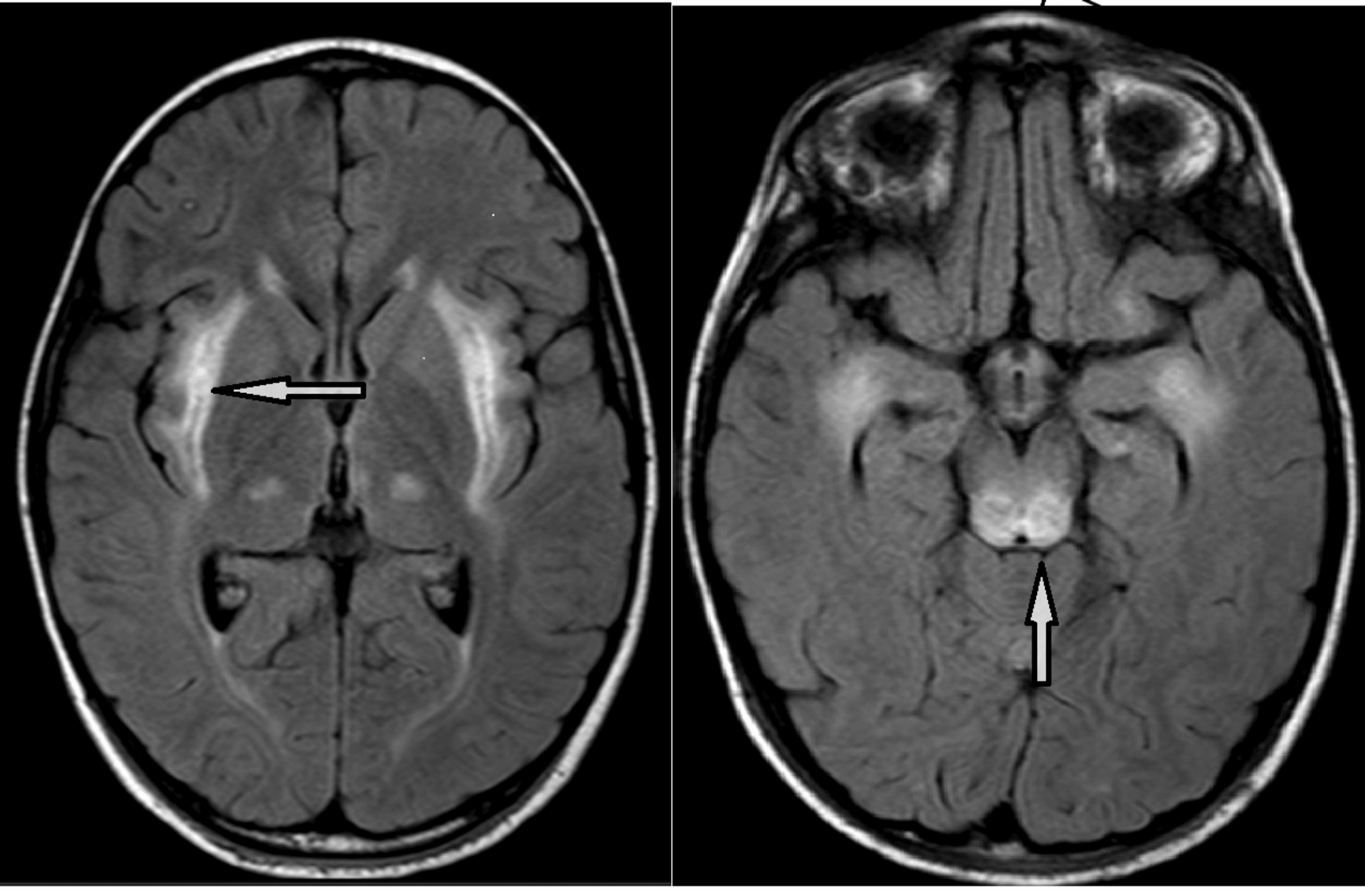
FLAIR axial images showing hyperintense signals in the external capsules, both thalami, dorsal pons, and hippocampi, with relative sparing of the basal ganglia. The arrow in the left-side MRI image indicates a hyperintense signal in the external capsule, while the arrow in the right-side image points to the signal in the dorsal pons. FLAIR: Fluid-attenuated inversion recovery

A seven-year-old boy, weighing 32 kg and a known case of ANE1, was scheduled for total hip reconstruction under general anesthesia. The child had been well until four years of age when he tested positive for influenza (H1N1) and later developed H1N1 meningitis. This progressed to acute disseminated encephalomyelitis. An MRI of the brain revealed a hyperintense signal in the external capsules, both thalami, dorsal pons, and hippocampi, with relative sparing of the basal ganglia, suggesting an infective or metabolic encephalopathy (Figure 1). A genetic workup revealed a monoallelic mutation of the RanBP2 gene. 

Upon further inquiry, it was noted that two of his siblings had died from meningitis. Over the next few years, he experienced multiple recurrences of ANE, which worsened his motor and cognitive function. He developed global developmental delay, poorly controlled seizures, a spastic-dystonic lower limb movement disorder, upper limb dystonic posturing, and right-sided hip dislocation. All his symptoms resulted in him being wheelchair-bound and dependent for all activities of daily living, with a Gross Motor Function Classification System (GMFCS) level of V. His medications included levetiracetam, clonidine, and clobazam, administered twice a day. In the past, he had undergone short diagnostic and surgical procedures, with a post-procedure admission to a high dependency unit (HDU), and these procedures were uneventful.

On clinical examination, he was wheelchair-bound, and his vitals were within normal limits. His cardiac and respiratory evaluations were unremarkable. His laboratory investigations, including liver, coagulation, and renal function tests, were within the normal range. Hemoglobin was 13.8 g/dl, and preoperative blood sugar was 103 mg/dl. His airway examination revealed a Mallampati score of 2, and there was no documented history of a difficult airway in the past.

The patient was premedicated with oral midazolam 0.4 mg/kg, 30 minutes before induction of anesthesia. ECG, blood pressure, oxygen saturation probe, and depth of anesthesia monitors were connected prior to the induction of anesthesia. Anesthesia was induced with a propofol bolus of 2 mg/kg and fentanyl 2 mcg/kg. He was paralyzed with cisatracurium 3 mg and intubated easily. Following induction, an arterial line was inserted, along with a 22 G cannula and a second large-bore IV cannula. Anesthesia was maintained with dexmedetomidine 0.2 mcg/kg/hr, remifentanil 0.2-0.3 mcg/kg/minute infusion, together with 0.4 minimal alveolar concentration (MAC) sevoflurane. Perioperatively, the patient received paracetamol 15 mg/kg and ketorolac 0.5 mg/kg. Plasmalyte was used intraoperatively to maintain hydration, and dextrose 10% was administered at 1-2 ml/kg/hr for glucose maintenance. Arterial blood gases were checked hourly, and the results were within normal limits. At the end of the surgery, the patient was extubated uneventfully and transferred to the post-anesthesia recovery unit before going to the general ward.

## Discussion

ANE1 is an extremely rare neurological condition, and information on the anesthetic management of such patients is limited. ANE1 has a pathological and clinical resemblance to other mitochondrial metabolic disorders, particularly Leigh’s disease. The management of ANE1 patients can be given the same perioperative considerations as these patients. This includes the maintenance of hydration, normoglycemia, normothermia, and acid-base balance, as well as the prevention of stress, catabolism, and infection. We maintained the patient on 2 ml/kg/hr of 10% dextrose to prevent hypoglycemia and did a blood gas analysis every hour to ensure that his blood sugar and acid-base balance were maintained within normal limits. Plasmalyte was used in place of Ringer's lactate for maintenance fluid, as lactate-containing fluids are prone to induce lactic acidosis and should be avoided. Temperature regulation and prevention of hypothermia were managed with the use of a fluid warmer and convective air warming blanket (Bair Hugger, 3M, United States).

One of the important considerations in the anesthetic management of patients with mitochondrial dysfunction is the possibility of propofol infusion syndrome. Propofol infusion syndrome is well described as a serious reaction in patients receiving long-term propofol infusion. The syndrome is characterized by severe lactic acidosis, followed by rhabdomyolysis and lipidemia, which can lead to cardiovascular collapse and death [16]. Propofol inhibits multiple electron transport chain complexes and acylcarnitine function in the mitochondria, resulting in an increase in serum acylcarnitines during prolonged infusions [17]. Multiple case reports suggest that patients with mitochondrial dysfunction are at risk of propofol infusion syndrome during prolonged exposure [18,19]. In our case, we maintained anesthesia with an infusion of dexmedetomidine and remifentanil with sevoflurane. Dexmedetomidine is considered safe for the anesthetic management of ANE patients [20]. Throughout the anesthesia procedure, anesthetic depth was monitored with the use of a Sedline Patient State Index (PSI) monitor (Sedline, Masimo Corp., United States).

Since ANE1 is known to get triggered by stress and infection, extreme care was taken to maintain an aseptic environment throughout the perioperative phase. We minimized the movement of staff in and out of the operating theatre and all aseptic precautions were taken throughout the procedure. The patient was given a stress dose of 50 mg hydrocortisone and also received two doses of antibiotic during the perioperative period. Table 1 summarizes the perioperative considerations for the management of patients with ANE.

**Table 1:** Suggested anesthetic considerations for acute necrotizing encephalopathy. Source: Authors' compilation

Anesthetic considerations for managing acute necrotizing encephalopathy	
Cardiac and respiratory evaluation	Preoperative cardiac and respiratory evaluation to rule out any underlying decompensation. Preoperative cardiac and respiratory optimization to improve post-procedure outcome.
Seizure management	Preoperative management of antiepileptic therapy. Documentation of therapeutic anticonvulsant levels. Normalize serum electrolytes and glucose. Midazolam has antiepileptic effects.
Acid-base and blood sugar management	Minimize preoperative fasting. Use dextrose-containing IV fluids for maintenance in the preoperative fasting period to provide basal glucose requirements. Avoid hypoglycemia in the perioperative period. Check preoperative lactate levels. Frequent intraoperative serum lactate, pH, and glucose monitoring. Avoid lactate-containing fluids (e.g., Ringer’s lactate). Monitor the depth of anesthesia and avoid long-acting opioids. Postoperative respiratory monitoring for major procedures. Maintain normothermia and normocapnia, and avoid any hypoxia.
Infection risk	Complete aseptic precautions. Proper antibiotic coverage.

## Conclusions

In conclusion, preoperative evaluation and optimization of the cardiac, neuromuscular, and respiratory systems are mandatory in patients with ANE1. Perioperatively, every effort must be made to maintain hydration, normoglycemia, and the prevention and correction of acid-base imbalance. Avoidance of lactate-containing fluids and perioperative stress, as well as the prevention and treatment of infection, are paramount.
